# When Surgery Won’t Cut It: Successful Management of Eruptive Squamous Atypia With Combination Medical Therapy

**DOI:** 10.7759/cureus.37694

**Published:** 2023-04-17

**Authors:** Sara Yumeen, Benjamin J Kahn, Alexandra Leonard, Asghar Shah, Abrar A Qureshi, Elie Saliba

**Affiliations:** 1 Dermatology, Warren Alpert Medical School of Brown University, Providence, USA; 2 Medicine, Brown University, Rhode Island, USA; 3 Dermatology/Epidemiology, Warren Alpert Medical School of Brown University, Providence, USA

**Keywords:** acitretin, 5-fluorouracil, intralesional, eruptive squamous atypia, multiple keratoacanthoma

## Abstract

The termeruptive squamous atypia(ESA) is used to describe squamous proliferations that do not present with high-grade histologic features and for which surgical management may exacerbate the condition. Non-surgical management of ESA with radiation, local or systemic chemotherapy, retinoids, or immunotherapy have been reported with variable success. In contrast, combination treatment with retinoids, immunomodulatory or chemotherapeutic agents may result in a more durable response. We report a case of recalcitrant *ESA* of the lower extremities where complete clinical remission was induced with triple combination medical management with intralesional 5-fluorouracil, field treatment with topical 5-fluorouracil and imiquimod, and oral acitretin. Our case adds to the literature supporting combination medical therapy for challenging cases of ESA.

## Introduction

Keratoacanthomas (KA) are typically considered to be low-to-moderate grade histologic variants of squamous cell carcinoma (SCC). Although classification and management remain contentious, complete surgical excision is considered the current gold standard treatment [1]. However, KAs have been reported to koebnerize by accidental or iatrogenic trauma, including trauma induced by surgical excision [2]. Recurrent trauma from biopsy and surgical treatment has been reported to lead to a cycle of tumor removal and recurrence, a condition that has been termed eruptive keratoacanthoma [2]. However, in 2019 Que et al. [2] proposed that these proliferations may be better described as eruptive squamous atypia (ESA). These lesions do not present with high-grade histologic features and managing them surgically may exacerbate the condition [2].

Literature review of this entity is challenging as cases have been reported as eruptive keratoacanthoma in cases when patients had multiple true SCCs KA-type and also in cases of ESA. Que et al. reported that ESA responded well to intralesional 5-fluorouracil (5-FU) with or without adjunct treatment with topical steroids, topical 5-FU, cryotherapy, or oral acitretin2. Non-surgical management with radiation or local or systemic chemotherapy, retinoids, and immunotherapy have been reported with variable success, however, they have often demonstrated incomplete resolution or recurrence with medical monotherapy [3-5]. In contrast, combination treatment with retinoids and immunomodulatory or chemotherapeutic agents may result in a more durable response [5,6].

We report a case of recalcitrant ESA of the lower extremities where complete clinical remission was induced with triple combination medical management with intralesional 5-fluorouracil, field treatment with topical 5-fluorouracil and imiquimod, and oral acitretin. Our case adds to the literature supporting combination medical therapy for challenging cases of ESA.

## Case presentation

A 68-year-old female with Fitzpatrick type I skin and past medical history of Grave’s disease status-post radiation, rheumatoid arthritis status-post treatment with rituximab, and IgG immunodeficiency presented for evaluation of numerous lower extremity lesions. She was initially seen at an outside practice six months prior to presentation where biopsies initially showed actinic keratoses with lichenoid inflammation but repeat biopsy was consistent with well-differentiated squamous cell carcinoma. The lesions were repeatedly treated with cryotherapy without improvement, and two lesions were resected, following which new lesions developed immediately post-operatively.

**Figure 1:**
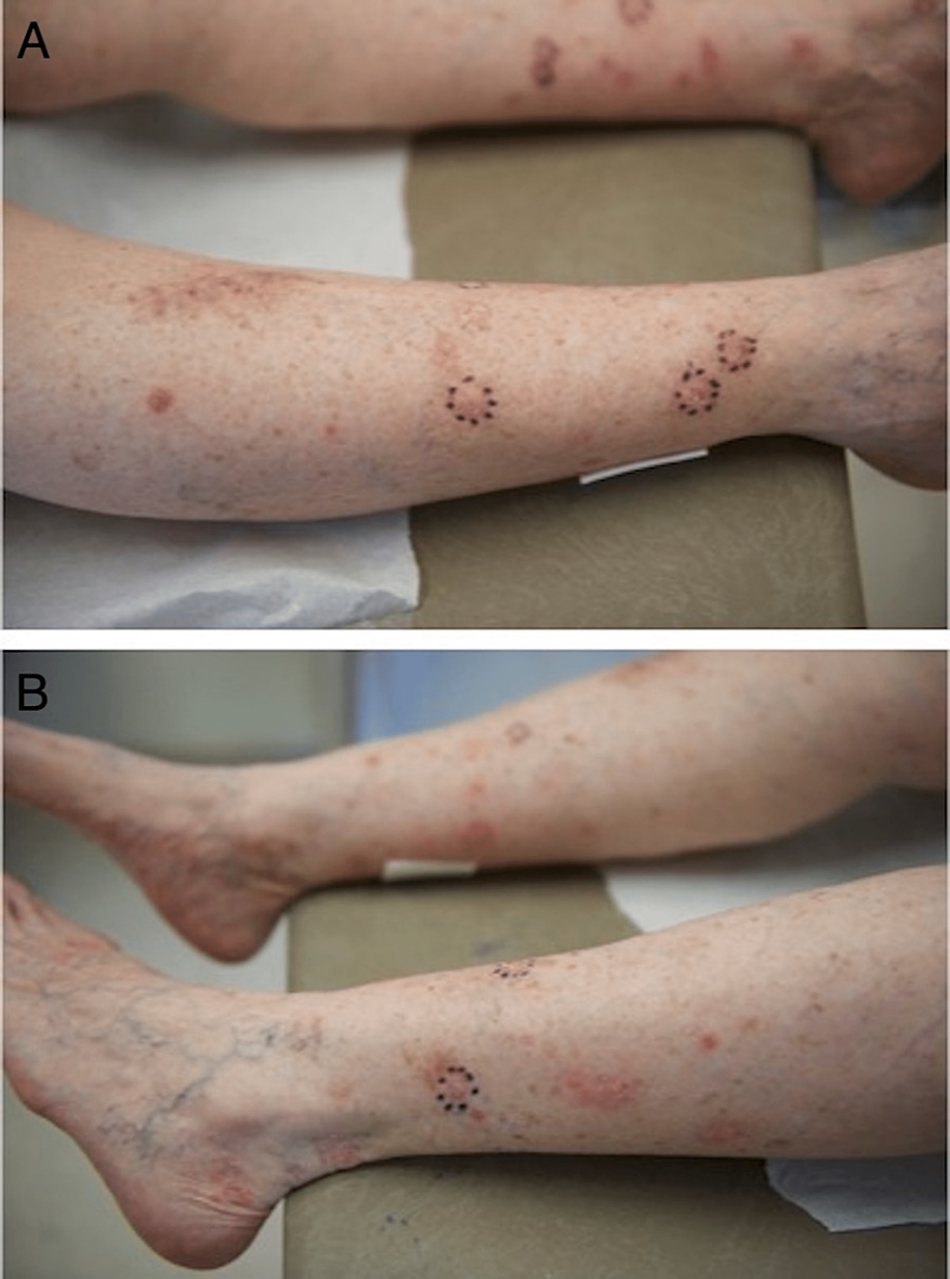
Patient image at the time of presentation A 68-year-old female presented with innumerable exophytic pink papules and plaques with central crust, in the range of 20-30 lesions, clinically consistent with keratoacanthomas or squamous proliferations on the bilateral lower extremities. Prominent lesions are outlined with a purple marking pen.

On presentation to our clinic, her legs were covered in innumerable exophytic pink papules and plaques with central crust, in the range of 20-30 lesions, clinically consistent with keratoacanthomas or squamous proliferations (Figures 1A-1B). We suspected eruptive squamous atypia induced by repeated biopsy, cryotherapy, and excision in the setting of background sun exposure. The patient’s history of IgG immunodeficiency and resultant immunocompromise may have also led to decreased immune surveillance and clearance of the proliferations.

**Figure 2:**
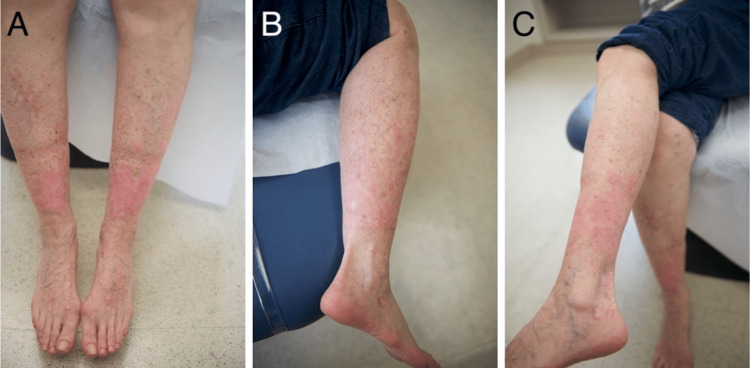
Patient images after the therapy Complete clinical remission was induced with triple combination medical management with intralesional 5-fluorouracil, field treatment with topical 5-fluorouracil and imiquimod, and oral acitretin.

The two largest lesions were injected with intralesional 5-fluorouracil (5-FU) and the patient was started on 10 mg of oral acitretin daily. On follow-up, the lesions treated with 5-FU demonstrated notable regression, so twelve more discrete and large lesions were treated with intralesional 5-FU. The patient was also instructed to field treat her legs with alternating courses of 5-FU and imiquimod for four to six weeks. The majority of her lesions had involuted on follow-up after this therapy (Figures 2A-2C). Acitretin was tapered­­­ due to her clinical improvement. Once her lower extremities had been clear for six months, acitretin was discontinued. The patient currently remains in clinical remission with full clinical resolution.

## Discussion

Patients with multiple hyperkeratotic proliferations with low-grade histologic features, particularly on the lower extremities in the setting of repeated trauma, are likely presenting with eruptive squamous atypia (ESA) [2]. These lesions may be misdiagnosed and treated surgically as though they are keratoacanthomas (KAs) or aggressive/recurrent squamous cell carcinoma (SCC). These surgical treatments are often ineffective and further biopsies and surgical treatment may lead to yet further development of additional lesions due to Koebnerization [2]. We describe a case of ESA where clinical remission was achieved through combination medical approach with intralesional 5-FU, topical 5-FU and imiquimod, and oral acitretin.

Medical management with topical steroids, topical or systemic retinoids, topical immunomodulators like imiquimod, or chemotherapeutic agents such as methotrexate, cyclophosphamide, and topical 5-FU has been reported for ESA and presumed eruptive KA [3-6]. Our approach utilizes a systemic retinoid (acitretin) in combination with a topical immunomodulator (imiquimod) and topical and intralesional chemotherapy (5-FU). These therapies have been reported with a variable response as monotherapy, but greater efficacy has been reported with combination therapy of retinoids with immunomodulatory or chemotherapeutic agents [4,5]. We postulate that our triple combination of acitretin, imiquimod, and 5-fluorouracil may be more efficacious as it provides treatment through multiple mechanisms. While the role of topical and oral retinoids in chemoprevention and treatment of skin cancers has not yet been elucidated, their effect is postulated to be due to their regulation of growth and differentiation of keratinocytes, and sensitization to apoptosis [7]. Imiquimod enhances tumor cell apoptosis while promoting immune response to the tumor, and 5-FU acts as a chemotherapeutic agent inducing cell death of malignant keratinocytes as an anti-metabolite inhibiting thymidylate synthase [8]. We hypothesize that targeting these pathways simultaneously induces both regression of clinically apparent lesions while preventing development of new proliferations.

## Conclusions

Our case supports current literature suggesting that a combination of medical therapies may be most effective when managing ESA, and care should be taken to avoid misdiagnosis and mistreatment of these lesions as though they are malignancies such as KA or SCC. Surgical treatments can exacerbate the condition without providing resolution, and we recommend a combination medical approach, as has been supported in the literature. To our knowledge, this is the first report of successful treatment with our triple combination of topical imiquimod, oral acitretin, and intralesional 5-fluorouracil.
